# 3D-QSAR, Molecular Docking, and MD Simulations of Anthraquinone Derivatives as PGAM1 Inhibitors

**DOI:** 10.3389/fphar.2021.764351

**Published:** 2021-11-25

**Authors:** Yuwei Wang, Yifan Guo, Shaojia Qiang, Ruyi Jin, Zhi Li, Yuping Tang, Elaine Lai Han Leung, Hui Guo, Xiaojun Yao

**Affiliations:** ^1^ College of Pharmacy, Shaanxi University of Chinese Medicine, Xianyang, China; ^2^ School of Pharmacy, Lanzhou University, Lanzhou, China; ^3^ Dr. Neher’s Biophysics Laboratory for Innovative Drug Discovery, Macau University of Science and Technology, Macau, China; ^4^ State Key Laboratory of Quality Research in Chinese Medicine, Macau University of Science and Technology, Macau, China

**Keywords:** PGAM1, molecular docking, molecular dynamics simulation, CoMFA, CoMSIA

## Abstract

PGAM1 is overexpressed in a wide range of cancers, thereby promoting cancer cell proliferation and tumor growth, so it is gradually becoming an attractive target. Recently, a series of inhibitors with various structures targeting PGAM1 have been reported, particularly anthraquinone derivatives. In present study, the structure–activity relationships and binding mode of a series of anthraquinone derivatives were probed using three-dimensional quantitative structure–activity relationships (3D-QSAR), molecular docking, and molecular dynamics (MD) simulations. Comparative molecular field analysis (CoMFA, r^2^ = 0.97, q^2^ = 0.81) and comparative molecular similarity indices analysis (CoMSIA, r^2^ = 0.96, q^2^ = 0.82) techniques were performed to produce 3D-QSAR models, which demonstrated satisfactory results, especially for the good predictive abilities. In addition, molecular dynamics (MD) simulations technology was employed to understand the key residues and the dominated interaction between PGAM1 and inhibitors. The decomposition of binding free energy indicated that the residues of F22, K100, V112, W115, and R116 play a vital role during the ligand binding process. The hydrogen bond analysis showed that R90, W115, and R116 form stable hydrogen bonds with PGAM1 inhibitors. Based on the above results, 7 anthraquinone compounds were designed and exhibited the expected predictive activity. The study explored the structure–activity relationships of anthraquinone compounds through 3D-QSAR and molecular dynamics simulations and provided theoretical guidance for the rational design of new anthraquinone derivatives as PGAM1 inhibitors.

## Introduction

Reprogramming energy metabolism has been regarded as one of the 10 essential hallmarks of cancer cells (Hanahan and Weinberg, 2011), which was called the “Warburg effect.” In 1924, Warburg found that cancer cells are more likely to metabolize glucose by means of aerobic glycolysis instead of oxidative phosphorylation as in normal cells (Wang et al., 2018a; Huang et al., 2019b). Cancer metabolic reprogramming is the performance of adapting to the environment during tumor formation or metastasis. More and more scientists are focusing on the pivotal enzymes in the metabolic reprogramming of cancer cells in order to find new cancer treatment targets (Wang et al., 2018b).

Phosphoglycerate mutase 1 (PGAM1) is a key enzyme that catalyzes the invertible conversion of 3-phosphoglycerate (3-PG) and 2-phosphoglycerate (2-PG) during the process of glycolysis (Fothergill-Gilmore and Watson, 1989). Recent studies have proven that once the expression of PGAM1 is upregulated, it will promote tumor cell proliferation and tumor growth in coordination with glycolysis and biosynthesis (Hitosugi et al., 2012). PGAM1 regulates the proliferation of cancer cells in term of biosynthesis regulation, partly by regulating intracellular levels of its product 2-PG and 3-PG (Hitosugi et al., 2012). In the oxidative pentose phosphate pathway (PPP), 3-PG inhibits 6-phosphogluconate dehydrogenase after binding, while 2-PG feedback control of the levels of through activates 3-phosphoglycerate dehydrogenase. In addition, PGAM1 is overexpressed in multiple cancers (Li and Liu, 2020), including ovarian cancer (Zhang et al., 2020), non–small-cell lung cancer (NSCLC) (Li et al., 2020), colorectal cancer (Liu et al., 2008; Lei et al., 2011), pancreatic ductal adenocarcinoma (PDAC) (Liu et al., 2018), prostate cancer (PCa) (Wen et al., 2018), and glioma (Xu et al., 2016). Particularly, high expression of PGAM1 was associated with poor prognosis in NSCLC patients (Sun et al., 2018; Li et al., 2020). Downregulation of the expression of PGAM1 or suppression of its metabolic activity will lead to weakened cell proliferation and tumor growth (Hitosugi et al., 2012; Peng et al., 2016; Liu et al., 2018). Thus, PGAM1 is considered to be an emerging target for cancer treatment.

Due to the important role of PGAM1 in the occurrence and development of tumors, many researchers have focused on the discovery and characterization of small molecules that can target and modulate the metabolic activity of PGAM1 (Huang et al., 2019a). MJE3 was first revealed as a covalent PGAM1 inhibitor on Lys 100 by the Cravatt group in 2005 (Evans et al., 2005). (-)-Epigallocatechin-3-gallate (EGCG) is a natural product extracted from green tea, which was first discovered as a non-substrate competitive PGAM1 inhibitor with potent inhibition activity against PGAM1 (Li et al., 2017). Anthraquinone derivatives PGMI-004A (Hitosugi et al., 2012) and xanthone derivatives (Wang et al., 2018b) were identified as allosteric PGAM1 inhibitors by the Zhou group, which exhibited moderate inhibition activity on PGAM1. As another anthraquinone derivative, HKB99 was identified to allosterically obstruct the activation of PGAM1, thereby affecting its catalytic activity and the intermolecular interaction of ACTA2 (Huang et al., 2019c; Liang et al., 2021). Based on the excellent anticancer activity of PGMI-004A and HKB99, new small molecules with the anthraquinone core have been synthesized, which may have similar mechanisms of action and therapeutic potential. Therefore, the design and development of novel small molecules with an anthraquinone core targeting PGAM1 may prove to be an effective strategy for the treatment of cancer cells.

Computer-aided drug design is an effective tool in the drug discovery and design process. It can not only be used to predict the activity of small molecules, explain the action mechanism, and provide guidance for the design of more effective drug molecules but also reduce the consumption of manpower and material resources (Jorgensen, 2004). To elucidate the structure–activity relationships and provide optimization guidance for anthraquinone derivatives, 62 collected compounds were employed to construct 3D-QSAR models using CoMFA and CoMSIA methods. According to the contour maps by 3D-QSAR and the crucial residues by MD simulations, 7 compounds with high predictive activity were designed. This study will provide a valuable theoretical basis for the activity prediction and structural modification of targeted PGAM1 inhibitors containing anthraquinone structures.

## Materials and Methods

### Data Sets and Preparation

In order to ensure the reliability of activity values and reduce accidental errors, a set of 78 PGAM1 inhibitors were retrieved from different literature sources in terms of the same group (Wang et al., 2018a; Wang et al., 2018b; Huang et al., 2019a; Huang et al., 2019b). The molecular structure and experimental bioactivity of all chemicals are listed in Table 1. First, corresponding IC_50_ values of experimental bioactivity expressed in nM were converted into negative logarithm (–lgIC_50_) and acted as the dependent variable for the QSAR modeling. According to the diversity of the molecular structure and activities, all compounds were split into a training set and a test set at a ratio of approximately 4:1. Finally, 62 compounds were selected randomly as the training set and the remaining 16 compounds as the test set. The molecular structure of each compound was determined using ChemDraw 18.0 and then imported to SYBYL 6.9 (SYBYL, XX) to minimize the energy based on the Tripos force field with a convergence criterion of 0.01 kcal/mol. The Gasteiger–Hückel method was employed to calculate the partial atomic charges. Then, the multisearch strategy was performed to obtain the lowest energy conformation, and the lowest energy geometry after being filled with energy was reserved for alignment.

**TABLE 1:** Structure and corresponding activity data of reported PGAM1 inhibitors.

Number	R_1_	R_2_	R_3_	R_4_	R_5_	R_6_	X	IC_50_ (μM)	pIC_50_
1	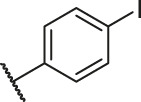	H	OH	H	H	H	O	10.10	5.00
2	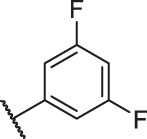	H	OH	H	H	H	O	13.20	4.88
3^a^	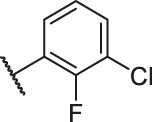	H	OH	H	H	H	O	6.40	5.19
4	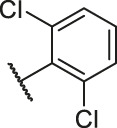	H	OH	H	H	H	O	10.2	4.99
5	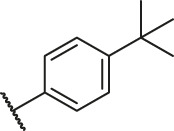	H	OH	H	H	H	O	8.40	5.08
6	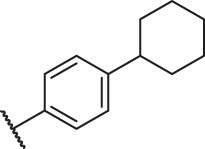	H	OH	H	H	H	O	5.90	5.23
7	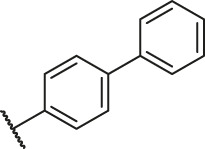	H	OH	H	H	H	O	5.50	5.26
8	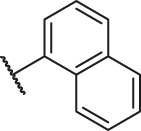	H	OH	H	H	H	O	6.00	5.22
9^a^	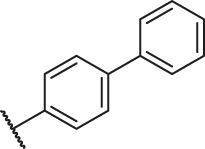	H	H	H	H	H	O	14.3	4.84
10	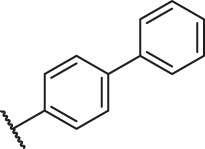	H	H	H	H	-OCH3	O	6.50	5.19
11^a^	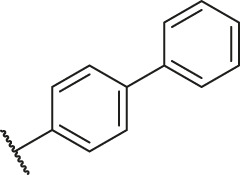	H	H	H	H	-CH3	O	8.60	5.07
12	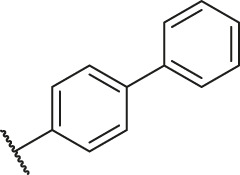	H	H	-OCH3	H	H	O	4.60	5.34
13	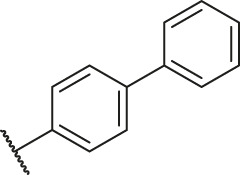	H	H	-CH3	H	H	O	8.00	5.10
14	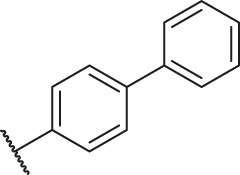	H	H	Cl	H	H	O	3.50	5.46
15	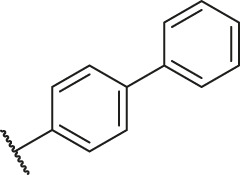	H	H	F	H	H	O	13.7	4.86
16	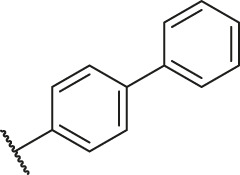	H	H	-NO2	H	H	O	2.10	5.68
17	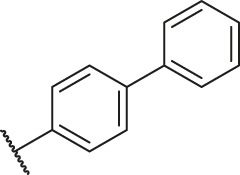	H	H	OH	H	H	O	6.40	5.19
18	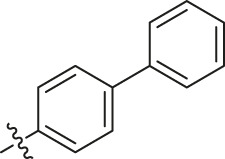	H	H	−COOCH3	H	H	O	2.70	5.57
19	-CH3	OH	H	H	H	H	-C=O	5.37	5.27
20	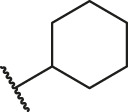	OH	H	H	H	H	-C=O	2.05	5.69
21	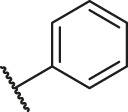	OH	H	H	H	H	-C=O	1.75	5.76
22	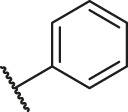	OH	H	H	H	H	-C=O	1.50	5.82
23^a^	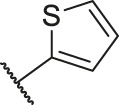	OH	H	H	H	H	-C=O	0.36	6.44
24	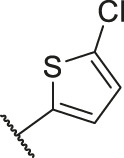	OH	H	H	H	H	-C=O	0.84	6.08
25	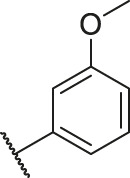	OH	H	H	H	H	-C=O	0.55	6.26
26	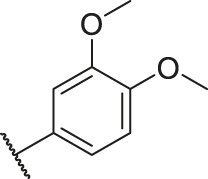	OH	H	H	H	H	-C=O	0.48	6.32
27	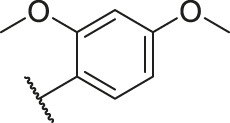	OH	H	H	H	H	-C=O	2.81	5.55
28	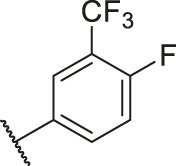	OH	H	H	H	H	-C=O	2.86	5.54
29	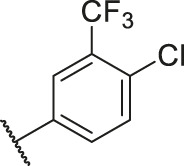	OH	H	H	H	H	-C=O	0.63	6.20
30	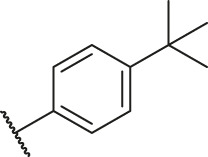	OH	H	H	H	H	-C=O	0.55	6.26
31	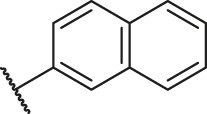	OH	H	H	H	H	-C=O	0.49	6.31
32^a^	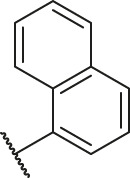	OH	H	H	H	H	-C=O	0.19	6.72
33^a^	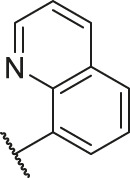	OH	H	H	H	H	-C=O	1.29	5.89
34	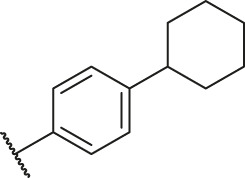	OH	H	H	H	H	-C=O	2.05	5.69
35	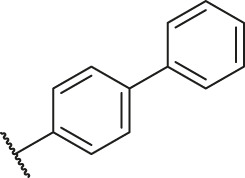	OH	H	H	H	H	-C=O	0.097	7.01
36	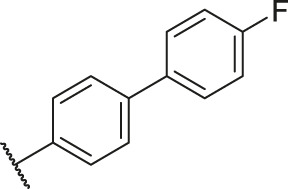	OH	H	H	H	H	-C=O	0.25	6.60
37^a^	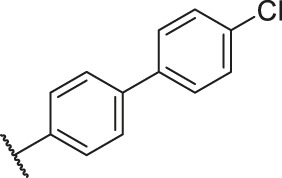	OH	H	H	H	H	-C=O	0.26	6.59
38	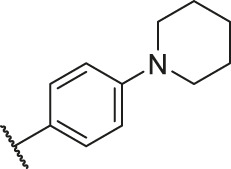	OH	H	H	H	H	-C=O	0.14	6.85
39	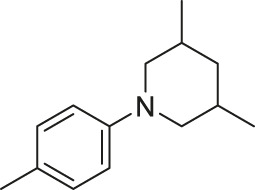	OH	H	H	H	H	-C=O	0.33	6.48
40	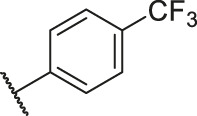	OH	H	H	H	H	-C=O	2.60	5.59
41	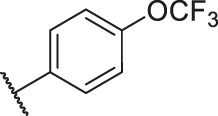	OH	H	H	H	H	-C=O	0.54	6.27
42	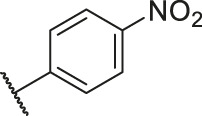	OH	H	H	H	H	-C=O	0.90	6.05
43	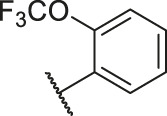	OH	H	H	H	H	-C=O	0.47	6.33
44	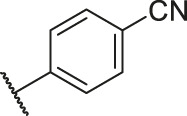	OH	H	H	H	H	-C=O	2.20	5.66
45^a^	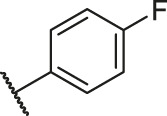	OH	H	H	H	H	-C=O	0.61	6.21
46	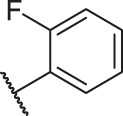	OH	H	H	H	H	-C=O	0.54	6.27
47	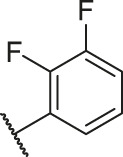	OH	H	H	H	H	-C=O	0.79	6.10
48^a^	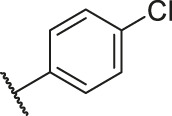	OH	H	H	H	H	-C=O	0.89	6.05
49	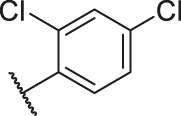	OH	H	H	H	H	-C=O	0.27	6.57
50	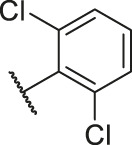	OH	H	H	H	H	-C=O	0.28	6.55
51	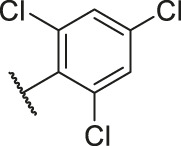	OH	H	H	H	H	-C=O	0.89	6.05
52	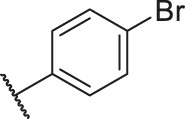	OH	H	H	H	H	-C=O	0.90	6.05
53	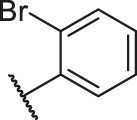	OH	H	H	H	H	-C=O	0.26	6.59
54	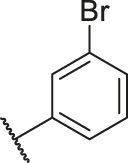	OH	H	H	H	H	-C=O	0.20	6.70
55	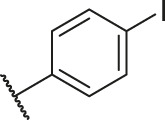	OH	H	H	H	H	-C=O	0.35	6.46
56	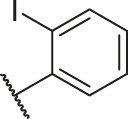	OH	H	H	H	H	-C=O	0.47	6.33
57	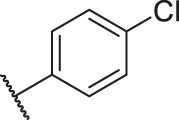	OH	H	H	H	H	-C=O	0.26	6.59
58	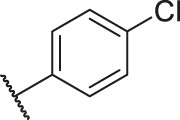	-OCH3	H	H	H	H	-C=O	2.92	5.53
59	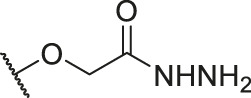	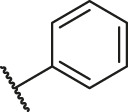	H	H	H	H	-C=O	2.00	5.70
60	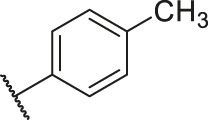	OH	OH	H	H	H	O	2.80	5.55
61	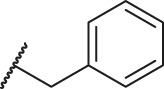	OH	OH	H	H	H	O	7.20	5.14
62	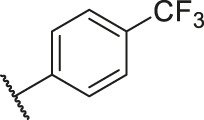	OH	OH	H	H	H	O	1.90	5.72
63	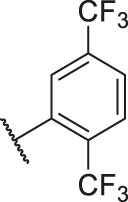	OH	OH	H	H	H	O	3.50	5.46
64	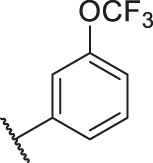	OH	OH	H	H	H	O	6.30	5.20
65^a^	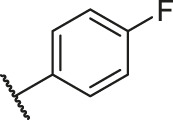	OH	OH	H	H	H	O	5.80	5.24
66	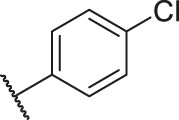	OH	OH	H	H	H	O	5.50	5.26
67	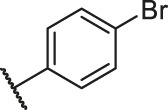	OH	OH	H	H	H	O	3.60	5.44
68^a^	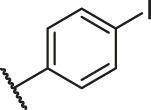	OH	OH	H	H	H	O	2.90	5.54
69^a^	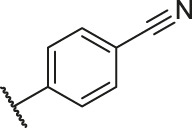	OH	OH	H	H	H	O	1.90	5.72
70^a^	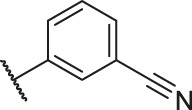	OH	OH	H	H	H	O	4.20	5.38
71^a^	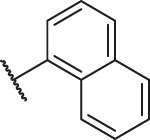	OH	OH	H	H	H	O	2.10	5.68
72	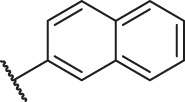	OH	OH	H	H	H	O	1.70	5.77
73	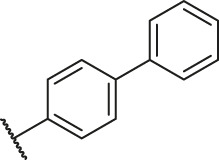	OH	OH	H	H	H	O	1.60	5.80
74	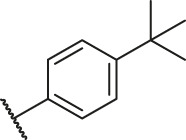	OH	OH	H	H	H	O	1.20	5.92
75	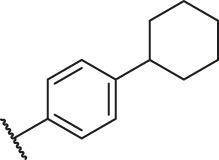	OH	OH	H	H	H	O	2.60	5.59
76^a^	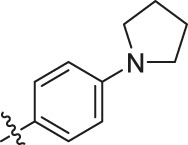	OH	OH	H	H	H	O	0.50	6.30
77^a^	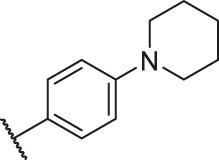	OH	OH	H	H	H	O	2.70	5.57
78	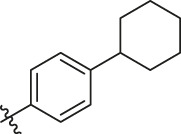	OH	OH	H	H	H	O	1.00	6.00

a Test set for the validation of the 3D-QSAR model.

### Molecular Alignment

Molecular alignment in terms of the same structure is considered to be one of the most significant elements in the process of built 3D-QSAR modeling. Hence, molecular alignment based on the most active molecule, 35, was employed by atom-by-atom fits. After a common substructure is set, the dominant conformations of the remaining 77 compounds are selected for superimposition.

### Construction of CoMFA and CoMSIA Models

The 3D-QSAR model for the training set compound was built after alignment by using SYBYL 6.9 software. The CoMFA (Cramer et al., 1988b) and CoMSIA (Cramer et al., 1988b) are the most widely used methods for constructing 3D-QSAR. The CoMFA and CoMSIA descriptors were obtained by placing the superposed compound in a 3D cubic lattice with a grid spacing of 2 Å. Using the SP^3^ hybrid carbon as the probe atom, the Lennard–Jones and the coulomb potential were applied to obtain the steric field energy and electrostatic field energy of each lattice point. The contributions of the hydrogen bond acceptor field, hydrogen bond donor field, and hydrophobic field were calculated by the probe atom. The partial least squares method (Cramer et al., 1988a) was employed to deal with the linear correlation between the CoMFA and CoMSIA fields and biological activity. The cross-validation correlation coefficient (q^2^) and optimum number of components (N) were obtained using the leave-one-out method for cross-validation analysis. In addition, the $r_{m}^{2}$ (Roy et al., 2013; Cardoso et al., 2016), $r_{pred}^{2}$ , external standard deviation error of prediction (SDEPext), and applicability domain (Roy et al., 2015; de Assis et al., 2016) were also calculated to evaluate the performance of built models.

### Evaluation of the 3D-QSAR Models

The predictive capabilities of built 3D-QSAR models were evaluated *via* the test set of 16 compounds. After all compounds were superimposed upon compound 35, the pIC_50_ values of all compounds were estimated through the built CoMFA and CoMSIA models.

### Molecular Docking

To obtain more accurate docking results, the resolution of all crystal structures of PGAM1 in complex with small molecules obtained from the RSCB Protein Data Bank (PDB) was compared, and 5Y35, with the best resolution of 1.99 Å, was preserved as the docking template. Subsequently, the Protein Preparation Wizard module within (Schrödinger, 2015) was utilized to preprocess the crystal structure, including adding hydrogens and side chains, deleting water molecules, and calculating partial charges and protonation states by using the OPLS2005 force field (Jorgensen et al., 1996). Then, a grid box centered at the native ligand with a similar size was produced to determine the binding pocket of PGAM1 by using the Grid Generation module of the Schrödinger package. All molecules were preprocessed using the LigPrep module implemented in the Schrödinger package, and the ionization states were calculated using Epik (Shelley et al., 2007) at pH = 7.0 ± 2.0. Finally, all chemicals were docked into the binding pocket of PGAM1 and evaluated using the standard precision (SP) mode of Glide. The scale factor was set at 0.8, and the partial charge intercept was set at 0.15. The 10,000 poses of each ligand during the initial docking phase were preserved for evaluation.

### Molecular Dynamics Simulations

To obtain the structural basis and significant residues involved in the process of ligand binding, molecular dynamics simulations were employed in terms of the crystal structure of compounds 23 and 49 using Amber16 (Case et al., 2005). The general AMBER force field (GAFF) (Wang et al., 2004) was employed to parameterize the compounds, while the AMBER ff14SB force field (Maier et al., 2015) was employed for the PGAM1 structure. The partial charges of compounds were calculated by using the restrained electrostatic potential fitting procedure (Bayly et al., 1993; Cieplak et al., 1995; Fox and Kollman, 1998) based on the electrostatic potentials calculated using the Hartree–Fock (HF) method with the 6-31G* basis set in the Gaussian 09 package (Frisch et al., 2009). Then, the complex was solvated in a cubic box of TIP3P waters, with the solute 10 Å away from the water box boundary. After adding sodium ions to neutralize each system, the steepest descent method followed by the conjugate-gradient method were employed to minimize the system every 2,500 steps. Subsequently, each system was heated in the NVT ensemble from 0 to 300 K in 50 ps restraint on backbone atoms. The restraint force was gradually decreased from 5 to 0.1 kcal/(mol Å^2^) within 0.9 ns. Under a periodic boundary condition, 50 ns MD simulations were performed at 300 K and 1 atm without any restraint. The particle mesh Ewald method (Linse and Linse, 2014) was used to calculate the long-range electrostatic interactions, and the SHAKE method (Ryckaert et al., 1977) was employed to constrain all covalent bonds containing hydrogen atoms.

### Trajectory Analysis

After the MD simulation finished, trajectories were dissected *via* the Cpptraj module (Roe and Cheatham, 2013) in AmberTools 16. First, the root mean square deviations (RMSDs) value was calculated in terms of the last 10 ns of each MD trajectory. Second, the molecular mechanics/generalized born surface area (MM/GBSA) approach (Massova and Kollman, 2000) was applied to calculate the binding free energy. After withdrawing a total of 2,500 snapshots, the MM/GBSA calculation was executed on each snapshot. The binding free energy (ΔG_bind_) was calculated as follows (Hou et al., 2011; Sun et al., 2014):

$$\Delta G_{\mathrm{bind}}=G_{\mathrm{complex}}-\left(G_{\mathrm{protein}}+G_{\mathrm{ligand}}\right)$$

where the energy term (G) is estimated as follows:

$$G=E_{vdw}+E_{ele}+G_{GB}+G_{GBSUR}$$

In the equations above, the *Evdw*, *Eele*, *G*_*GB*_, and *G*_GBSUR_ represent van der Waals, electrostatic energy, the electrostatic contribution to the solvation free energy, and non-polar contribution to the solvation free energy, respectively. The changes of conformational entropy were ignored. Moreover, the total free energy was decomposed to each residue in PGAM1 to obtain the crucial residues contributed to the ligand binding process.

## Results and Discussion

### CoMFA and CoMSIA Models

**FIGURE 1:**
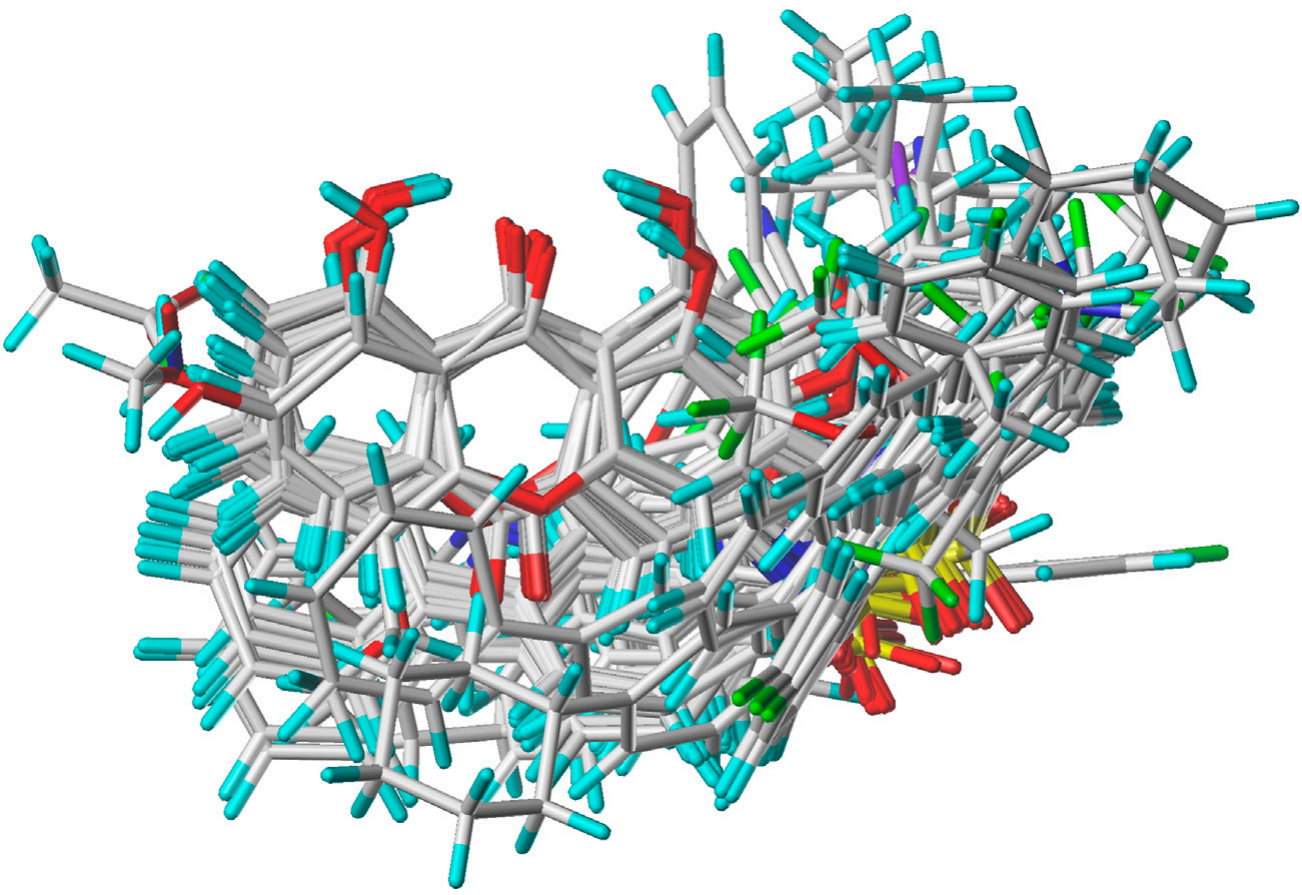
Structural alignment of all the molecules in the training set based on the common substructure of compound 35.

In the present study, a series of 78 PGAM1 inhibitors were obtained. The molecular structures and pIC_50_ values of all molecules are listed in Table 1. The quality of molecular superposition is considered to be one of the important factors affecting 3D-QSAR prediction accuracy (Cho et al., 1996). On the basis of the structure and bioactivity of PGAM1 inhibitors, the compounds in the training set were aligned to compound 35, which had the highest activity based on the common substructure. It can be seen from Figure 1 that the common skeleton of all molecules is overlapped. However, the side chains of several compounds surround the common skeleton due to the large difference. Then, the 3D-QSAR models of CoMFA and CoSIA were successfully developed.

To examine the predictive ability and reliability of the built model, q^2^ and r^2^ were applied to evaluate the predictive power of the built 3D-QSAR model, r^2^, F, and SEE values were employed to assess the reliability of the model, and $r_{m}^{2}$ , $r_{pred}^{2}$ , and SDEP_ext_ values were utilized for external validation of the model. Table 2 lists the classical parameter statistics of CoMFA and CoMSIA models. In general, r^2^ > 0.7 and q^2^, $r_{m}^{2}$ , $r_{pred}^{2}$ >0.5 are necessary for a good model (Pratim Roy et al., 2009). As shown in Table 2, the values of q^2^, N, SEE, r^2^, $r_{m}^{2}$ , $r_{pred}^{2}$ , SDEP_ext_, and F are 0.81, 6, 0.106, 0.97, 0.78, 0.89, 0.22, and 258.06, respectively. The results show that the built CoMFA model exhibits a good stability and predictive ability. The contribution of the steric field and the electrostatic field is 81 and 19%, respectively, indicating that the biological activity of compounds is more affected by the steric field. In addition, the predicted activity of the new chemical is only valid when the predicted compound falls within the applicability domain of the developed model (Roy et al., 2015). The calculated results show that all compounds are within the application domain of the built CoMFA model, so this prediction result is reliable.

**TABLE 2:** Summary of CoMFA and CoMSIA models.

PLS statistics	CoMFA	CoMSIA
q^2^	0.81	0.82
N	6	6
r^2^	0.97	0.96
F	258.06	228.71
$r_{m}^{2}$	0.78	0.79
$r_{pred}^{2}$	0.89	0.89
SDEP_ext_	0.22	0.23
SEE	0.11	0.11
Steric	0.81	0.20
Electrostatic	0.19	0.22
Hydrophobic	-	0.40
Hydrogen bond acceptor	-	0.18

Different field combinations of CoMSIA models were constructed, and it had been proved that CoMSIA-SEHA is the best model. Based on this model, the values of q^2^, N, SEE, r^2^, $r_{m}^{2}$ , $r_{pred}^{2}$ , SDEP_ext_, and F are 0.82, 6, 0.11, 0.96, 0.79, 0.89, 0.23, and 228.71, respectively. In this model, the contribution of the steric field is 20%, that of the electrostatic field is 22%, that of the hydrophobic field is 40%, and that of the hydrogen bond acceptor field is 18%, respectively. The results show that the hydrophobic field has a greater effect on the bioactivity of the PGAM1 inhibitors. The calculation results of the application domain show that almost all the compounds are within the application domain of the CoSIA model, except for compound 24 with an S_new_ of 3.87 and compound 25 with an S_new_ of 4.06. By analyzing the descriptors in CoMSIA, we found that compounds 24 and 25 have the largest electrostatic field contribution. The experimental and predicted values of the biological activity of the training set and the test set in the established CoMFA and CoMSIA models are shown in Table 3.

**TABLE 3:** Experimental pIC_50_ (Exp.), predicted pIC_50_ (Pred.), and corresponding residuals (Res.) of the anthraquinone derivatives.

Number	pIC50	CoMFA		CoMSIA	
	Exp	Pred	Res	Pred	Res
1	5.00	5.00	0.00	5.02	0.03
2	4.88	5.00	0.12	5.05	0.17
3	5.19	4.98	−0.21	5.14	−0.05
4	4.99	4.94	−0.05	5.11	0.12
5	5.08	5.11	0.04	5.11	0.03
6	5.23	5.34	0.11	5.15	−0.08
7	5.26	5.28	0.02	5.31	0.05
8	5.22	5.16	−0.06	5.18	−0.05
9	4.84	5.33	0.49	5.29	0.44
10	5.19	5.18	−0.01	5.29	0.10
11	5.07	5.26	0.19	5.21	0.15
12	5.34	5.35	0.01	5.24	−0.10
13	5.10	5.27	0.17	5.31	0.21
14	5.46	5.34	−0.12	5.29	−0.17
15	4.86	4.94	0.08	4.84	−0.03
16	5.68	5.78	0.10	5.67	−0.01
17	5.19	5.23	0.03	5.17	−0.02
18	5.57	5.38	−0.19	5.51	−0.05
19	5.27	5.46	0.19	5.31	0.04
20	5.69	5.61	−0.08	5.74	0.05
21	5.76	5.70	−0.06	5.74	−0.02
22	5.82	5.84	0.02	5.76	−0.07
23	6.44	6.33	−0.12	6.32	−0.13
24	6.08	6.09	0.01	5.88	−0.20
25	6.26	6.44	0.18	6.26	0.00
26	6.32	6.38	0.06	6.24	−0.08
27	5.55	5.59	0.03	5.48	−0.07
28	5.54	5.54	−0.01	5.46	−0.08
29	6.20	6.21	0.01	6.25	0.04
30	6.26	6.27	0.01	6.33	0.07
31	6.31	6.35	0.04	6.42	0.11
32	6.72	6.61	−0.11	6.51	−0.21
33	5.89	5.96	0.07	6.45	0.56
34	5.69	5.65	−0.04	5.69	0.00
35	7.01	7.08	0.07	6.97	−0.04
36	6.60	6.69	0.09	6.53	−0.07
37	6.59	6.84	0.26	6.80	0.21
38	6.85	6.84	−0.01	6.82	−0.03
39	6.48	6.48	0.00	6.66	0.18
40	5.59	5.61	0.03	5.53	−0.06
41	6.27	6.19	−0.07	6.33	0.06
42	6.05	6.08	0.03	6.13	0.08
43	6.33	6.31	−0.02	6.39	0.06
44	5.66	5.77	0.12	5.53	−0.12
45	6.21	6.21	0.00	6.38	0.17
46	6.27	6.43	0.16	6.37	0.10
47	6.10	6.09	−0.01	6.14	0.04
48	6.05	6.05	0.00	6.11	0.06
49	6.57	6.53	−0.04	6.49	−0.08
50	6.55	6.36	−0.19	6.40	−0.16
51	6.05	6.13	0.08	6.24	0.19
52	6.05	6.04	0.00	6.32	0.27
53	6.59	6.54	−0.05	6.51	−0.08
54	6.70	6.50	−0.20	6.49	−0.21
55	6.46	6.11	−0.34	6.39	−0.07
56	6.33	6.49	0.16	6.41	0.08
57	6.59	6.55	−0.03	6.44	−0.15
58	5.53	5.39	−0.15	5.56	0.02
59	5.70	5.69	−0.01	5.72	0.02
60	5.55	5.40	−0.15	5.52	−0.03
61	5.14	5.33	0.19	5.47	0.33
62	5.72	5.72	−0.01	5.72	0.00
63	5.46	5.43	−0.03	5.46	0.00
64	5.20	5.17	−0.03	5.21	0.01
65	5.24	5.52	0.29	5.48	0.24
66	5.26	5.34	0.08	5.10	−0.16
67	5.44	5.33	−0.12	5.48	0.04
68	5.54	5.34	−0.20	5.47	−0.07
69	5.72	5.39	−0.33	5.69	−0.04
70	5.38	5.41	0.03	5.11	−0.27
71	5.68	5.31	−0.37	5.63	−0.04
72	5.77	5.78	0.01	5.70	−0.07
73	5.80	5.78	−0.02	5.75	−0.05
74	5.92	5.92	0.00	5.88	−0.04
75	5.59	5.51	−0.08	5.52	−0.07
76	6.30	6.30	0.00	6.27	−0.03
77	5.57	5.39	−0.18	5.57	0.00
78	6.00	5.96	−0.04	6.01	0.01

**FIGURE 2:**
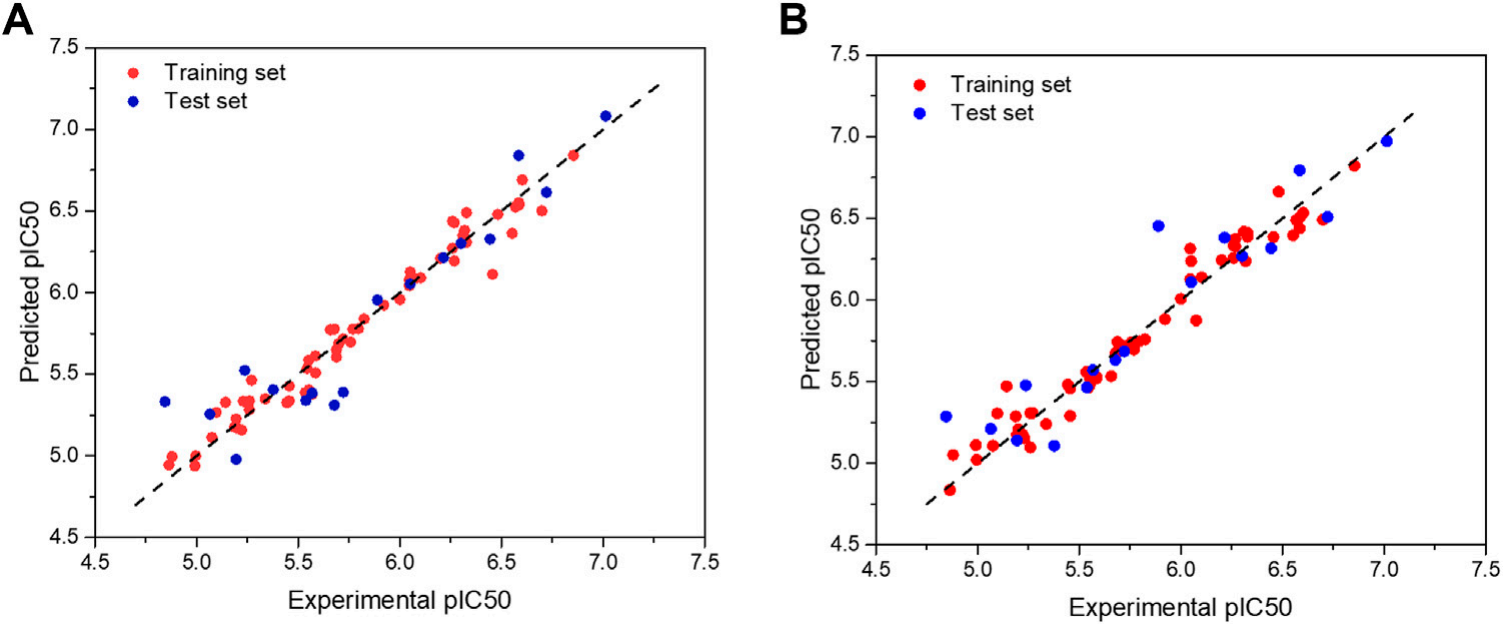
Scatter plot of experimental and predicted bioactivity values (pIC_50_)of the CoMFA **(A)** and CoMSIA models **(B)**, respectively.

The scatter plot of the experimental and predicted values of the studied PGAM1 inhibitor is shown in Figure 2. It can be seen from Figure 2 that the experimental and predicted bioactivity values of all molecules are distributed around the Y = X equation, indicating that the predicted values are in good accord with the experimental values, which further demonstrates that the model has good predictive ability.

### Contour Maps Analysis of CoMFA and CoMSIA

**FIGURE 3:**
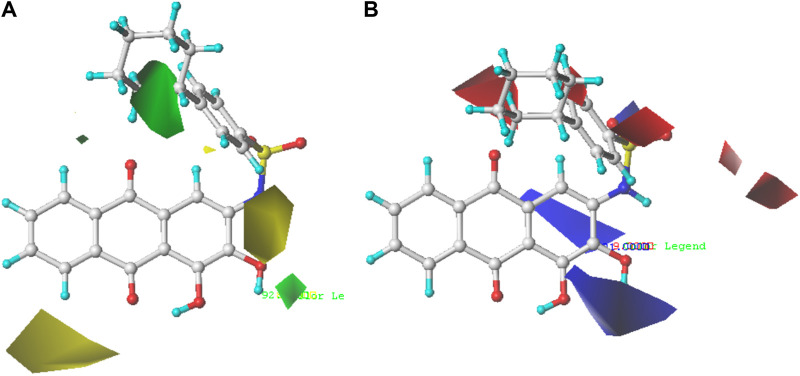
Steric contour map **(A)** and electrostatic contour map **(B)** of the CoMFA model based on molecule 35. Green regions represent bulky groups that increase anti-PGAM1 activity, while yellow regions represent sterically unfavored regions. Blue regions show where positive groups are beneficial for increasing anti-PGAM1 acitivity, and red regions show where negative groups are favored.

**FIGURE 4:**
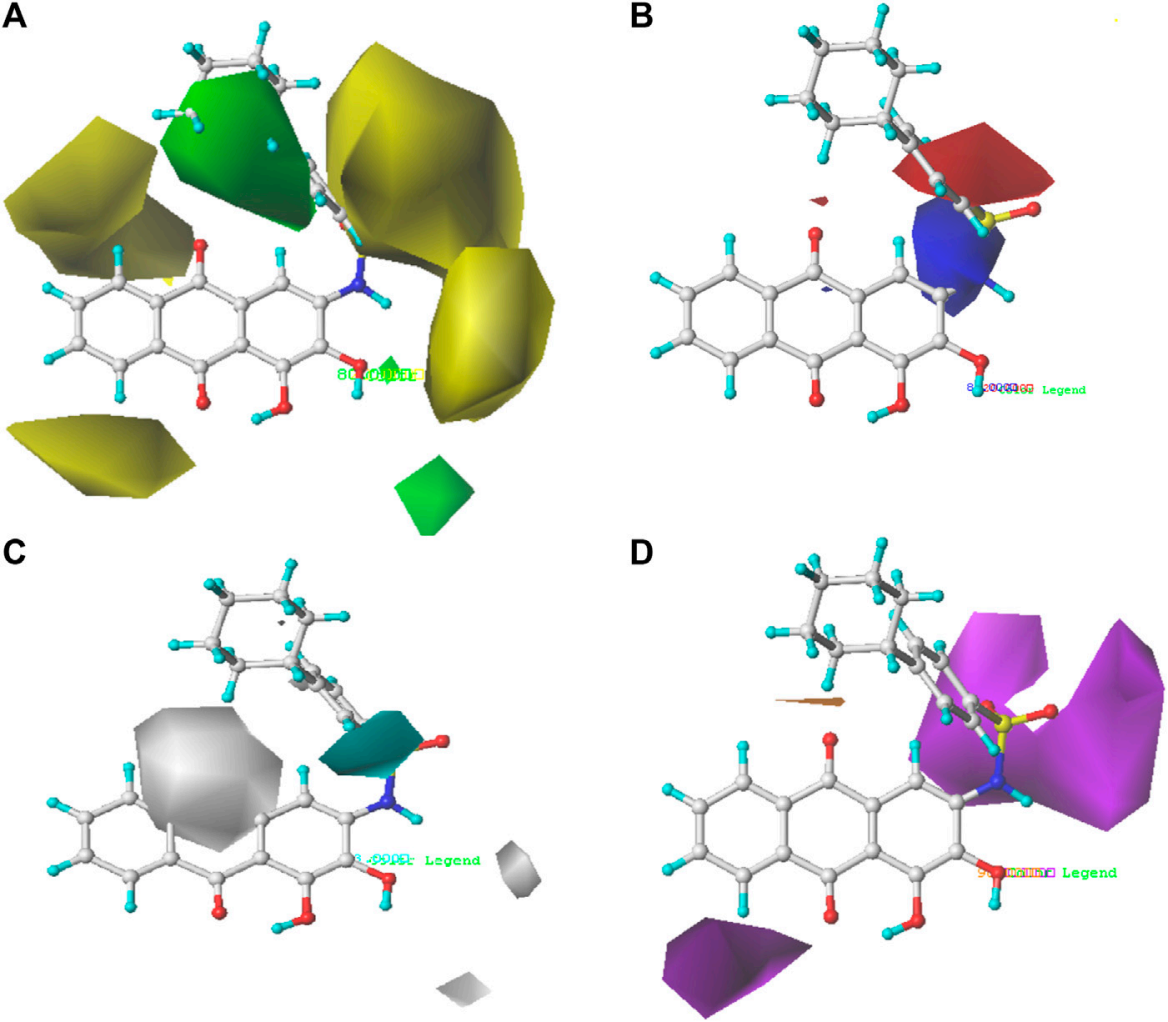
Steric contour map **(A)**, electrostatic contour map **(B)**, hydrophobic contour map **(C)**, and hydrogen bond acceptor contour map **(D)** of the CoMSIA model based on molecule 35. Green regions are sterically favored regions, while yellow regions are sterically unfavored regions. Blue regions are where electron-donating groups are favored, and red regions are where electron-withdrawing groups are favored. The cyan regions are where the hydrophobic group is favorable to the activity, while the white regions are where the hydrophilic group is favorable to the activity.

The structure–activity relationships between PGAM1 inhibitors and activity can be well demonstrated by using 3D contour maps to display the QSAR equation. The field type Stdev* Coeff was used to generate 3D contour maps. As shown in Figures 3, 4, compound 35 with the best anti-PGAM1 activity was selected as the template compound to dissect the results of CoMFA and CoMSIA models.

The contour map of the steric field of CoMFA is shown in Figure 3A, and the effect of the steric field on the activity is shown in green and yellow. The presence of green regions around the molecule indicates that the group with a large connecting space contributes to increasing the activity of the compound, while the presence of yellow regions indicates that the group with a large connecting space may decrease the activity of the compound. As can be seen from Figure 3A, there is a green region distributed on the R_1_ substituent, so the introduction of a slightly larger volume of groups at the R_1_ substituent site is conducive to the improvement of the activity of the compound. For example, compound 22 (pIC_50_ = 5.82) with a benzene ring was significantly higher than compound 19 (pIC_50_ = 5.27) in bioactivity. The contour map of the electrostatic field of CoMFA is shown in Figure 3B, and the effect of the electrostatic field on the activity is shown in blue and red. The blue regions around the molecule indicate that the connection of the electron-donating group is beneficial to the improvement of the activity of the compound, while the red regions indicate that the connection of the electron-withdrawing group is beneficial to the improvement of the activity of the compound. From Figure 3B, we can see that the connection of electron-withdrawing groups near the R_1_ substituent is conducive to improving the activity of the compound, so it can explain how the activity of compound 22 (pIC_50_ = 5.82) is higher than that of compound 19 (pIC_50_ = 5.27). There is a blue region around the R_2_ substituents of anthraquinone, where the introduction of electron groups is beneficial. For example, the bioactivity of compound 72 (pIC_50_ = 5.77) with a hydroxyl group was significantly higher than that of compound 8 (pIC_50_ = 5.22).

The contour map of the steric field (Figure 4A) and the electrostatic field (Figure 4B) of the CoMSIA is very similar to the CoMFA model, so they will not be explained here. The contour map of the hydrophobic field of the CoMSIA model is shown in Figure 4C. The cyan regions represent how the introduction of the hydrophobic group is favorable to the activity, while the white regions represent how the introduction of the hydrophilic group is favorable to the activity. There is a cyan region near the R_1_ substituent, indicating that the introduction of the hydrophobic group is very helpful to the improvement of the activity. Therefore, the biological activity of compound 22 (pIC_50_ = 5.82) is higher than that of compound 19 (pIC_50_ = 5.27). The contour map of the hydrogen bond receptor field of CoMSIA is shown in Figure 4D. The orange area is where the hydrogen bond acceptor group is conducive to the activity of the compound, and the purple area is where the hydrogen bond donor group is conducive to the activity of the compound. As shown in Figure 4D, there are purple regions with substituents of R_6_ and R_2_, where hydrogen bond donors can be imported to improve the anti-PGAM1 activity of the chemical. Moreover, a large purple region is near the nitrogen atom on the amino group, suggesting that the group may be a hydrogen bond donor.

**FIGURE 5:**
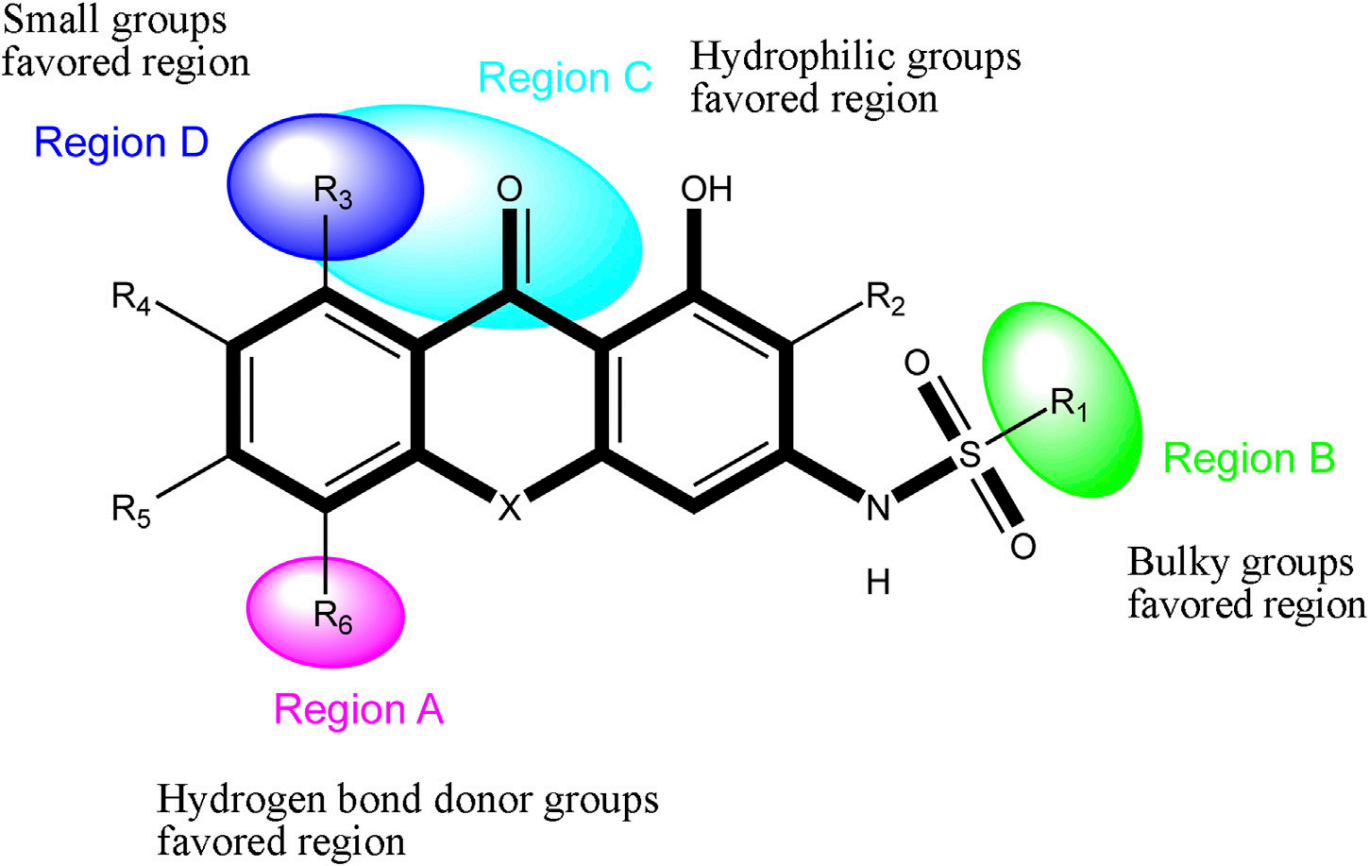
Structure–activity relationship diagram of anthraquinone PGAM1 inhibitors.

Based on the outcome of CoMFA and CoMSIA analysis, we obtained the structure–activity relationship diagram of anthraquinone compounds (see Figure 5). The introduction of hydrogen bond donors in Region A is beneficial to improving the activity of the compounds, such as the carbonyl group. The group with a large space in Region B is conducive to the activity of the compounds, such as biphenyl or p-cyclohexylbenzene (Huang et al., 2019b). The introduction of the hydrophilic group in Region C is conducive to the activity, such as hydroxyl groups (Wang et al., 2018a). The group with a small space in Region D can improve the activity of the compound, such as hydrogen.

### Molecular Docking Analysis

**FIGURE 6:**
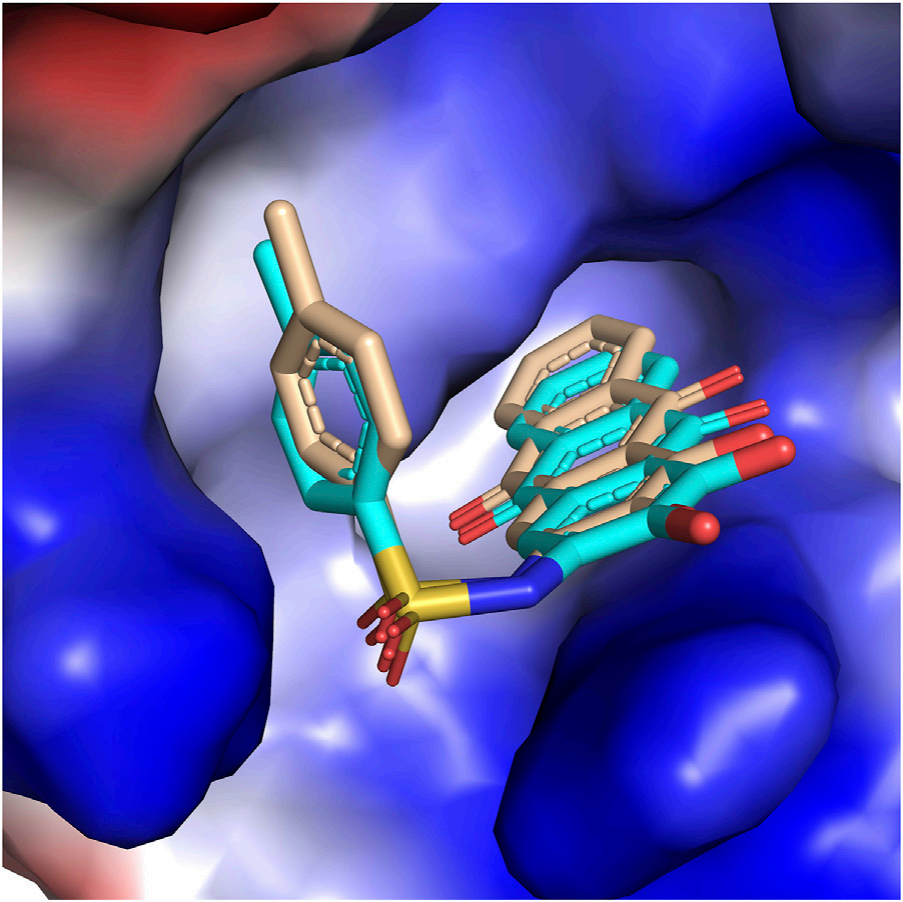
Surface of PGAM1 and docking pose of the native ligand based on the alignment. The yellow and cyan carbon atoms represent the native ligand and the docking pose, respectively.

The molecular docking method was employed to interpret the 3D-QSAR result and study the structural basis between PGAM1 and inhibitors. First, the reliability of the glide docking algorithm with the SP mode was evaluated by redocking analysis. It can be seen from Figure 6 that the redocking conformations of the molecule are well superimposed with the initial structure in PGAM1 protein. The RMSD value between docking conformation and native conformation is 0.005Å. The results suggest that the glide algorithm exhibits a good performance for the PGAM1 protein, which can reproduce the binding pose of the native ligand. Subsequently, all chemicals were docked into the binding site of PGAM1. However, we discover that the docking scores of these compounds are not correlated with the inhibitory activity, and the r^2^ of pIC_50_ vs. the docking score is 0.051, which demonstrates the fact that glide docking is not appropriate for all compounds. We speculate that one of the most important reasons is that 3-PG plays an important role in the process of compounds binding to PGAM1, and the glide scoring function currently used is not suitable for this system. In addition, because PGAM1 catalyzes the conversion of 3-PG to 2-PG in the physiological process, the current docking simulation methods cannot completely simulate this process. Therefore, the docking score and activity do not show a correlation.

### Molecular Dynamics Simulations

**FIGURE 7:**
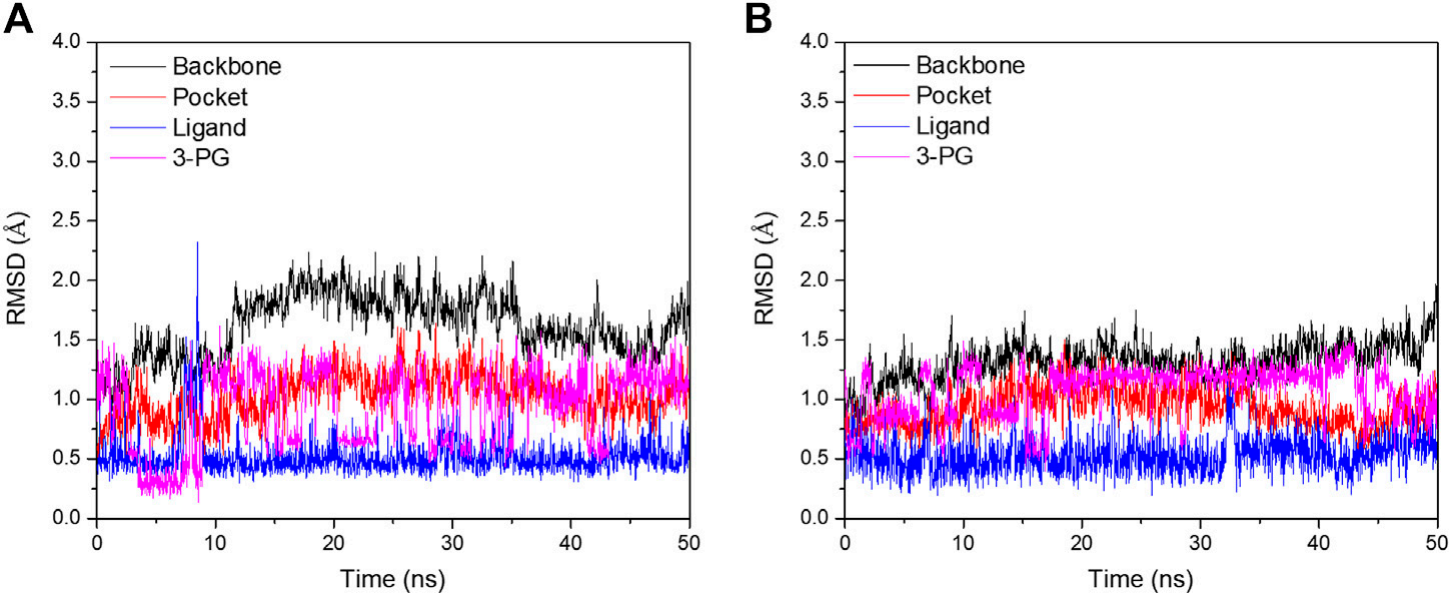
Fluctuation of RMSD values for two complexes during 50 ns MD simulation.

In order to further analyze the atomic details of the interaction between small molecules and PGAM1, molecular dynamics simulations were employed based on the co-crystal complex of compounds 23 (PDB ID: 5Y35) and 49 (PDB ID: 6ISN) using Amber 16, respectively. 50 ns simulation was performed for each complex. The RMSD plots of Cα, residues within the range of ligand 5Å, ligand, and 3-PG for complexes were shown in Figure 7. By monitoring the fluctuation of RMSDs, it can be found that the RMSD fluctuation of each system after 20 ns are all within the range of 2Å. Moreover, the fluctuation of binding free energy over time was also monitored. As shown in Supplementary Figure S1, binding free energy of each system fluctuates around 30 kcal/mol after 35 ns. In summary, these results indicate that the two systems finally reached a stable state.

During the process of small molecules binding to PGAM1, the hydrogen bond plays an important role as one of the most important non-bonding interactions. In order to explore the interaction between small molecules and PGAM1, the changes of the hydrogen bond between each residue of PGAM1 and the inhibitor were also monitored. The fraction of the hydrogen bond is greater than 10% as listed in Table 4. The results show that two hydrogen bonds formed between compounds 23 and 49 and Arg116, and the total occupancies are 180.12% and 38.48%, respectively. The results indicate that the hydrogen bonds formed between Arg116 of PGAM1 and inhibitors play a remarkable role in the binding of molecules. Besides, another hydrogen bond is also formed between compound 23 and Arg90 with the occupancy of 12.14%. It is precisely because the small molecules form hydrogen bonds with Arg116 and Arg90 to fix the anthraquinone skeleton of the compounds that compounds 23 and 49 are stably binding with PGAM1.

**TABLE 4:** Changes of the hydrogen bond over the MD simulations.

Complex	Donor	Acceptor	Occupancy (%)	Distance (Å)	Angle (°)
PGAM1-Compound 23	Arg116@N-H	Ligand@O5	75.08	2.93	152.74
	Arg116@NE-H	Ligand@O5	59.12	3.11	144.82
	Arg116@NE-H	Ligand@N1	45.92	3.24	152.12
	Arg90@N-H	Ligand@O1	12.24	3.12	130.43
PGAM1-Compound 49	Arg116@N-H	Ligand@O1	20.20	2.96	148.26
	Arg116@NE-H	Ligand@O1	18.28	3.05	147.35

### Binding Free Energy Calculation

The binding free energy is used as a reference standard for evaluating the activity of molecules. It is generally believed that the lower the binding value, the more stable the complex formed by the protein and the small molecule. To evaluate the binding affinity of each complex, the MM/GBSA method was performed to calculate the binding free energy of inhibitors. It can be seen from Table 5 that the binding free energy of compounds 23 and 49 are −27.40 kcal/mol and −27.85 kcal/mol, respectively, which are consistent with their biological activities. Among them, van der Waals energies (ΔE_vdw_) are −38.68 kcal/mol and −41.63 kcal/mol, respectively, and their values are much lower than other energy terms, indicating that hydrophobic interaction is the major contributor to the ligand binding process. In addition, electrostatic energy (ΔE_ele_) also contributes significantly to the binding free energy, which indicates that electrostatic interaction also plays a vital role in ligand binding. It is worth noting that the polar contribution (ΔG_GB_) is not conducive to ligand binding, which may be attributed to the large size of the binding pocket and the exposure of the hydrophobic ligand to the solvent.

**TABLE 5:** Calculated binding energy (kcal/mol) of inhibitor binding to PGAM1.

Terms	PGAM1-Compound 23	PGAM1-Compound 49
ΔE_ele_	−26.51 ± 7.59	−20.76 ± 6.23
ΔE_vdw_	−38.68 ± 2.92	−41.63 ± 3.31
ΔG_gas_	−65.19 ± 8.35	−62.39 ± 8.01
ΔG_GB_	41.62 ± 5.97	38.35 ± 5.21
ΔG_GBSUR_	−3.82 ± 0.16	−3.81 ± 0.15
ΔG_sol_	37.79 ± 5.91	34.55 ± 5.14
ΔG_bind_	−27.40 ± 4.21	−27.85 ± 3.68

ΔG_gas_ = ΔE_ele_ + ΔE_vdw_.

ΔG_sol_ = ΔG_GB_ + ΔG_GBSUR_.

ΔG_bind_ = ΔG_gas_ + ΔG_sol_.

**FIGURE 8:**
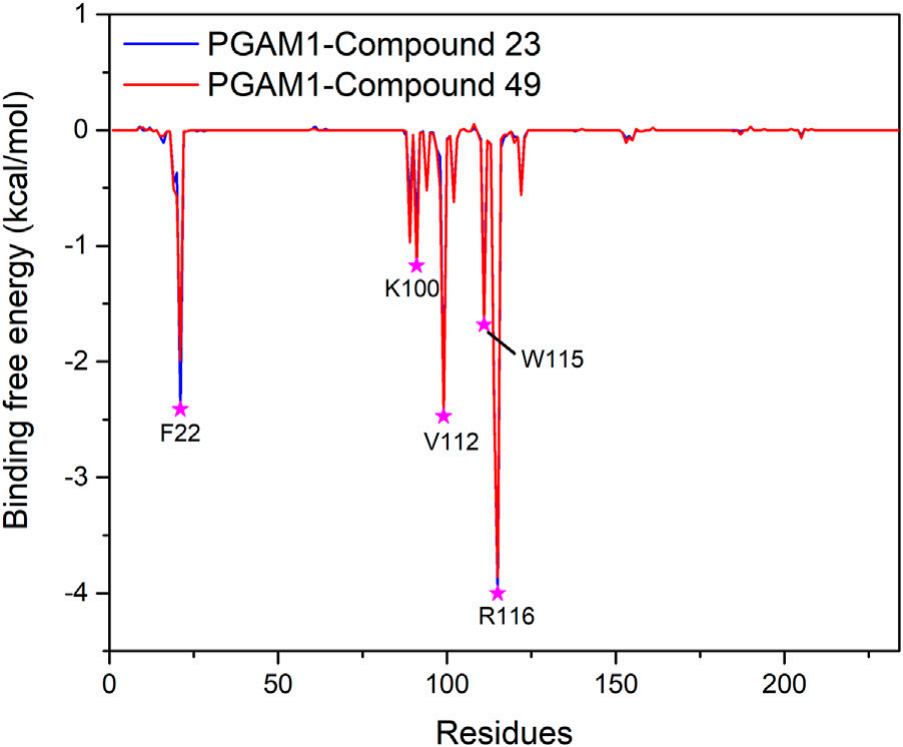
Binding free energy decomposition plots for the two systems.

**FIGURE 9:**
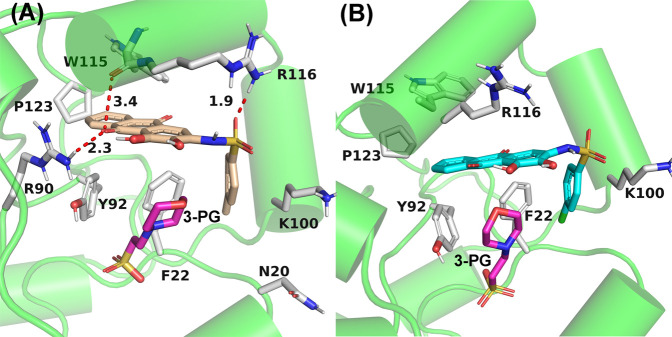
Average structures of PGAM1 with compounds 23 **(A)** and 49 **(B)**. The bonds of residues and ligands are represented in stick, and the carbon atoms of compound 23, compound 49, and residues are represented in yellow, cyan, and white, respectively. The red dotted line represents the hydrogen bond.

To further confirm the key residues referred to in the ligand binding process, MM/GBSA calculation was performed to decompose the binding free energy into inhibitor–residue pairs. It can be seen from Figure 8 that the primary residues with binding free energy less than −1 kcal/mol contributing to the ligand binding are F22, K100, V112, W115, and R116. In order to further observe the orientation of compounds and the position of the key residues, we extracted the average structure (see Figure 9). It can be seen from Figure 9 that compounds 23 and 49 adopt a similar binding pose, which is surrounded by those critical residues. Compound 23 forms three hydrogen bonds with R90, W115, and R116. Among the three of them, R90 and R116 show higher fraction in hydrogen bond analysis, while the bond length of W115 is 3.4 Å due to weak potency. For compound 49, there is no hydrogen bond formed between compound 49 and key residues, which may be due to the low occupancy.

### Design New PGAM1 Inhibitors

According to the structure–activity relationships obtained from CoMFA and CoMSIA models, seven molecules with the anthraquinone skeleton were designed as potential PGAM1 inhibitors by introducing new substituents at different positions of compound 35 (see Table 6). Compounds 79 and 80 were designed by adding the hydrogen bond donor in the R_6_ position to form the key hydrogen bond. Compounds 81, 82, and 83 were designed by introducing the substituent in the R_1_ position to increase volume. Based on the contribution of the steric and hydrogen bond donor, compounds 84 and 85 were designed. The pIC_50_ values of designed compounds were predicted by built CoMFA and CoMSIA models. As shown in Table 6, all of the designed compounds exhibit better inhibitory activity targeting PGAM1 than compound 35, and the predictive values are in accordance with the summarized structure–activity relationships.

**TABLE 6:** Newly designed PGAM1 inhibitors and the corresponding predicted activity value.

Number	R_1_	R_2_	R_3_	R_4_	R_5_	R_6_	X	CoMFA	CoMSIA
79	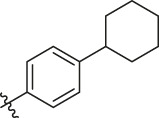	OH	H	H	H	OH	-C=O	7.07	7.03
80	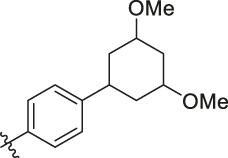	OH	H	H	H	NH2	-C=O	7.05	6.99
81	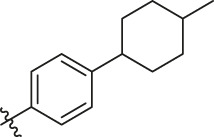	OH	H	H	H	H	-C=O	7.16	6.83
82	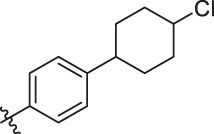	OH	H	H	H	H	-C=O	7.14	7.07
83	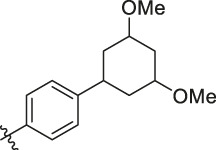	OH	H	H	H	H	-C=O	7.10	7.03
84	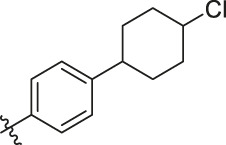	OH	H	H	H	OH	-C=O	7.14	6.85
85	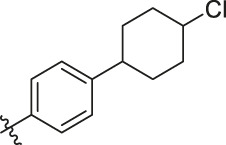	OH	H	H	H	OH	-C=O	7.13	7.00

## Conclusion

In the present study, a combined strategy of 3D-QSAR, molecular docking, and molecular dynamics simulations was applied to explore the structure–activity relationships of anthraquinone analogs. The built CoMFA (q^2^ = 0.81, r^2^ = 0.97, $r_{m}^{2}$ = 0.78, $r_{pred}^{2}$ = 0.89) and CoMSIA (q^2^ = 0.82, r^2^ = 0.96, $r_{m}^{2}$ = 0.79, $r_{pred}^{2}$ = 0.89) models have achieved satisfactory results in terms of the statistical results. The results show that the built models have good internal and external predictive power. The acquired contour maps elaborate the structure–activity relationships of anthraquinone derivatives and successfully predict the activity of the test set. According to the results of contour maps, the introduction of hydrogen bond donors in Region A, the group with a large space in Region B, the hydrophilic group in Region C, and the group with a small space in Region D could improve the activity of the compounds. The calculated results of binding free energy suggest that van der Waals interaction is the major contributor to the ligand binding process. The decomposition binding free energy and hydrogen bond show that small molecules with the anthraquinone core mainly interact with F22, R90, K100, V112, W115, and R116 of PGAM1. Based on these findings, 7 new compounds with the anthraquinone core were designed, and the predicted results show that all of the designed compounds exhibit great inhibitory activity against PGAM1. The constructed 3D-QSAR model will provide theoretical guidance for improving the activity of anthraquinone derivatives and help to develop inhibitors with potent anti-PGAM1 activity.

## Data Availability

The original contributions presented in the study are included in the article/Supplementary Material; further inquiries can be directed to the corresponding authors.
