# Supplementary material for: 3D-QSAR, Molecular Docking, and MD Simulations of Anthraquinone Derivatives as PGAM1 Inhibitors
Source: Front Pharmacol. 2021 Nov 25;12:764351. doi: 10.3389/fphar.2021.764351 (PMC8656170; doi:10.3389/fphar.2021.764351)
Supplement: Supplementary file 1 [file DataSheet1.docx]

Figure S1. The fluctuation of binding free energy over time for compounds 23 and 49.
